# Dietary protein load affects the energy and nitrogen balance requiring liver glutamate dehydrogenase to maintain physical activity

**DOI:** 10.1016/j.jbc.2024.107473

**Published:** 2024-06-13

**Authors:** Karolina Luczkowska, Yan Zhou, Angela M. Ramos-Lobo, Thierry Brun, Pierre Maechler

**Affiliations:** Department of Cell Physiology and Metabolism, University of Geneva Medical Center, Geneva, Switzerland

**Keywords:** liver, dietary protein, gluconeogenesis, ammonia, glutamate dehydrogenase

## Abstract

Provision of amino acids to the liver is instrumental for gluconeogenesis while it requires safe disposal of the amino group. The mitochondrial enzyme glutamate dehydrogenase (GDH) is central for hepatic ammonia detoxification by deaminating excessive amino acids toward ureagenesis and preventing hyperammonemia. The present study investigated the early adaptive responses to changes in dietary protein intake in control mice and liver-specific GDH KO mice (Hep-*Glud1*^−/−^). Mice were fed chow diets with a wide coverage of protein contents; *i.e.*, suboptimal 10%, standard 20%, over optimal 30%, and high 45% protein diets; switched every 4 days. Metabolic adaptations of the mice were assessed in calorimetric chambers before tissue collection and analyses. Hep-*Glud1*^−/−^ mice exhibited impaired alanine induced gluconeogenesis and constitutive hyperammonemia. The expression and activity of GDH in liver lysates were not significantly changed by the different diets. However, applying an *in situ* redox-sensitive assay on cryopreserved tissue sections revealed higher hepatic GDH activity in mice fed the high-protein diets. On the same section series, immunohistochemistry provided corresponding mapping of the GDH expression. Cosinor analysis from calorimetric chambers showed that the circadian rhythm of food intake and energy expenditure was altered in Hep-*Glud1*^−/−^ mice. In control mice, energy expenditure shifted from carbohydrate to amino acid oxidation when diet was switched to high protein content. This shift was impaired in Hep-*Glud1*^−/−^ mice and consequently the spontaneous physical activity was markedly reduced in GDH KO mice. These data highlight the central role of liver GDH in the energy balance adaptation to dietary proteins.

Our daily protein turnover is subjected to three interconnected fluxes of amino acids: dietary protein intake, *de novo* synthesis, and release of amino acids by proteolysis. The availability of energy substrates, in particular glucose, influences the net protein utilization and the catabolism of amino acids. Upon starvation or prolonged physical exercise, proteolysis in skeletal muscles promotes transport of amino acids, mainly as alanine and glutamine, from the peripheral tissues to the liver. This favors gluconeogenesis to maintain hepatic glucose production and euglycemia once glycogen stores are exhausted, with the accompanying consequence of increased production of ammonia (NH_3_) and the accumulation of its ammonium cation NH_4_^+^ (1). The latter situation can also be prompted by the ingestion of a high-protein diet, which stimulates catabolism and oxidation of amino acids that gives rise to substantial load of NH_4_^+^ (2). High dietary protein intake, or Paleolithic diet, is for instance favored by individuals to improve glucose tolerance in patients with type 2 diabetes (3). Hyperammonemia may be toxic for the brain as it alters neurotransmission and both clearance and recycling of neurotransmitter, an effect witnessed by liver failures such as hepatic encephalopathy (4).

Safe disposal of ammonia is carried out by dedicated hepatic metabolism, namely the formation of urea and the synthesis of glutamine, each molecule agreeing two nontoxic forms of nitrogen. Accordingly, the liver plays a crucial role in the adaptation to whole-body nitrogen balance and protein turnover. Regarding the zonation of these liver detoxifying pathways, there is an exclusive distribution of ornithine cycle enzymes required for ureagenesis in the periportal hepatocyte subpopulation, while glutamine synthetase (GS, *Glul*) is mostly present in the perivenous region. Glutamate dehydrogenase (GDH, *Glud1*) plays a key role in each of these ammonia disposal systems and is homogenously distributed throughout the liver (5). Zones with urea synthesis and GS activities are anatomically aligned one after the other. First, ureagenic periportal hepatocytes are supplied with blood enriched in circulating amino acids, and downstream come the glutamine-synthesizing cells incorporating leftover nitrogen into glutamine. This reflects the functional organization with periportal low-affinity and high-capacity urea system followed by perivenous high-affinity and low-capacity glutamine synthesis (6). Therefore, most of the blood ammonia from the portal circulation is metabolized in the periportal zone into urea for kidney excretion, the remaining being scavenged by fine tuning perivenous hepatocytes for glutamine synthesis (7).

Urea synthesis must be under dynamic control to either prevent the loss of valuable amino acids when their availability is scarce or to activate the ornithine cycle upon extensive amino acid catabolism as it represents the main way of elimination of their toxic byproduct NH_4_^+^. Due to the responsiveness of this system, daily dietary variations can be handled to avoid potentially toxic high circulating ammonia levels (8). Beside urea, the liver can generate glutamine as a complementary molecular sink for ammonia disposal. Blood glutamine concentrations vary according to the different dietary protein intakes, although indirectly through kidney function that catabolizes glutamine to secrete ammonia into urine and maintain acid-base homeostasis (7, 9).

The metabolism of amino acids involves GDH and aminotransferases for which glutamate and α-ketoglutarate are reaction partners. The transfer of an amino group to α-ketoglutarate produces glutamate which in turn can be used by GDH or aspartate aminotransferase (AST). GDH deaminating reaction provides ammonia, and AST transaminates oxaloacetate to produce aspartate, both products being necessary for urea generation. Importantly, GDH and aminotransferases are catalyzing reversible reactions, which enables hepatic nitrogen metabolism to tailor substrate concentrations to ornithine cycle capacity. GDH is an enzyme acting at the interface of carbohydrate and amino acid metabolism, thus bridging protein to glucose pathways (10). The bidirectional reaction catalyzed by GDH allows either the synthesis of glutamate or conversely its oxidation with the concomitant generation of ammonium (11, 12). GDH is allosterically activated by ADP and inhibited by GTP (13).

The present study addresses the role of liver GDH in the handling of ammonia upon short-term variations in dietary protein intake, with consequences on gluconeogenesis and rapid metabolic adaptations. We investigated the hepatic responses to amino acids, both *in vitro* and *in vivo*, under different metabolic conditions in liver-specific GDH KO mice. Furthermore, we challenged whole-body nitrogen metabolism and energy partitioning in mice fed with various regimens, going from suboptimal dietary protein intake to Paleolithic–like high protein diets.

## Results

### Hepatic GDH is mandatory for alanine-induced gluconeogenesis

**Figure 1:**
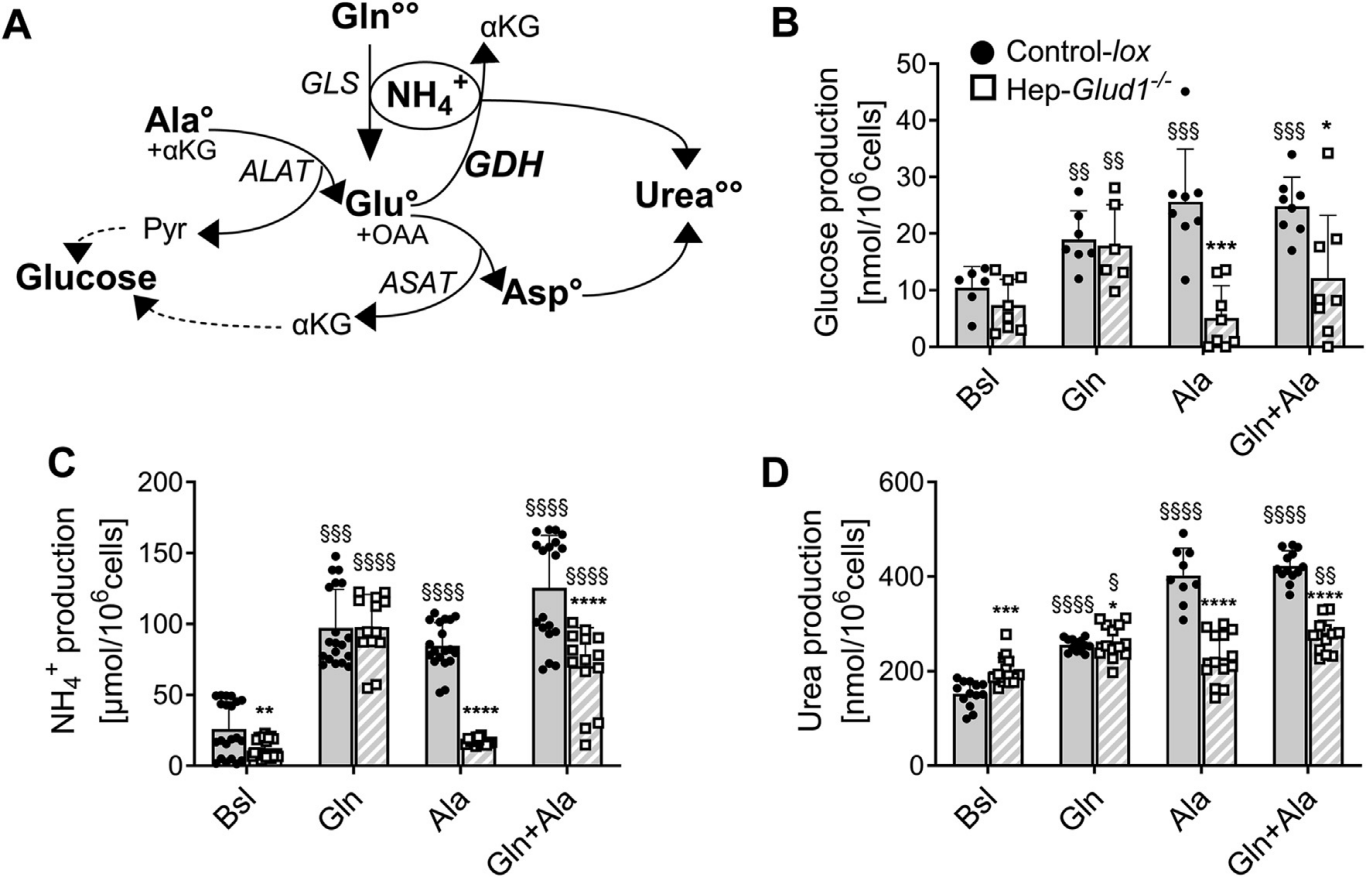
**Hepatocytes lacking GDH exhibit altered gluconeogenic alanine metabolism.** Cultured hepatocytes isolated from Control-*lox* and Hep-*Glud1*^*−/−*^ mice were stimulated with gluconeogenic amino acids. *A*, scheme presenting major biochemical pathways associated with amino acid gluconeogenesis and urea cycle (Gln, glutamine; Ala, alanine; Asp, aspartate; Pyr, pyruvate; Glu, glutamate; αKG, α-ketoglutarate; OAA, oxaloacetate; and °, N carried by the molecule). *B*, glucose production (n = 8), (*C*) ammonia production (n = 13–21), and (*D*) urea production (n = 10–15) in the nonstimulated basal state (Bsl) or stimulated with 5 mM Gln, 5 mM Ala, and a mix of both at 5 mM (Gln+Ala). Values are means ± SD; ∗*p* < 0.05, ∗∗*p* < 0.01, ∗∗∗*p* < 0.001, ∗∗∗∗*p* < 0.0001 *versus* Control-*lox*; §*p* < 0.05, §§*p* < 0.01, §§§*p* < 0.001, §§§§*p* < 0.0001 *versus* basal condition of the corresponding genotype. GDH, glutamate dehydrogenase.

Alanine and glutamine are physiological substrates for gluconeogenesis in the fasting state. It is assumed that glutamine-driven gluconeogenesis takes place predominantly in the kidneys and the intestine, while alanine is preferentially used for hepatic gluconeogenesis. Whatever the predominant substrate, this pathway requires the removal of the amino group (−NH2), coupled with ureagenesis in the liver (Fig. 1*A*). Cultured hepatocytes isolated from control mice (Control-*lox*) and mice lacking liver GDH (Hep-*Glud1*^*−/−*^) were stimulated with alanine, glutamine, or both amino acids. In control hepatocytes, all conditions resulted in the production of glucose, of the ammonium cation NH_4_^+^, and of urea (Fig. 1, *B–D*). In GDH null hepatocytes, the alanine response was fully abrogated, while the glutamine effects were all preserved; suggesting that the latter amino acid could contribute to liver gluconeogenesis and partially compensate for a lack of alanine metabolism.

**Figure 2:**
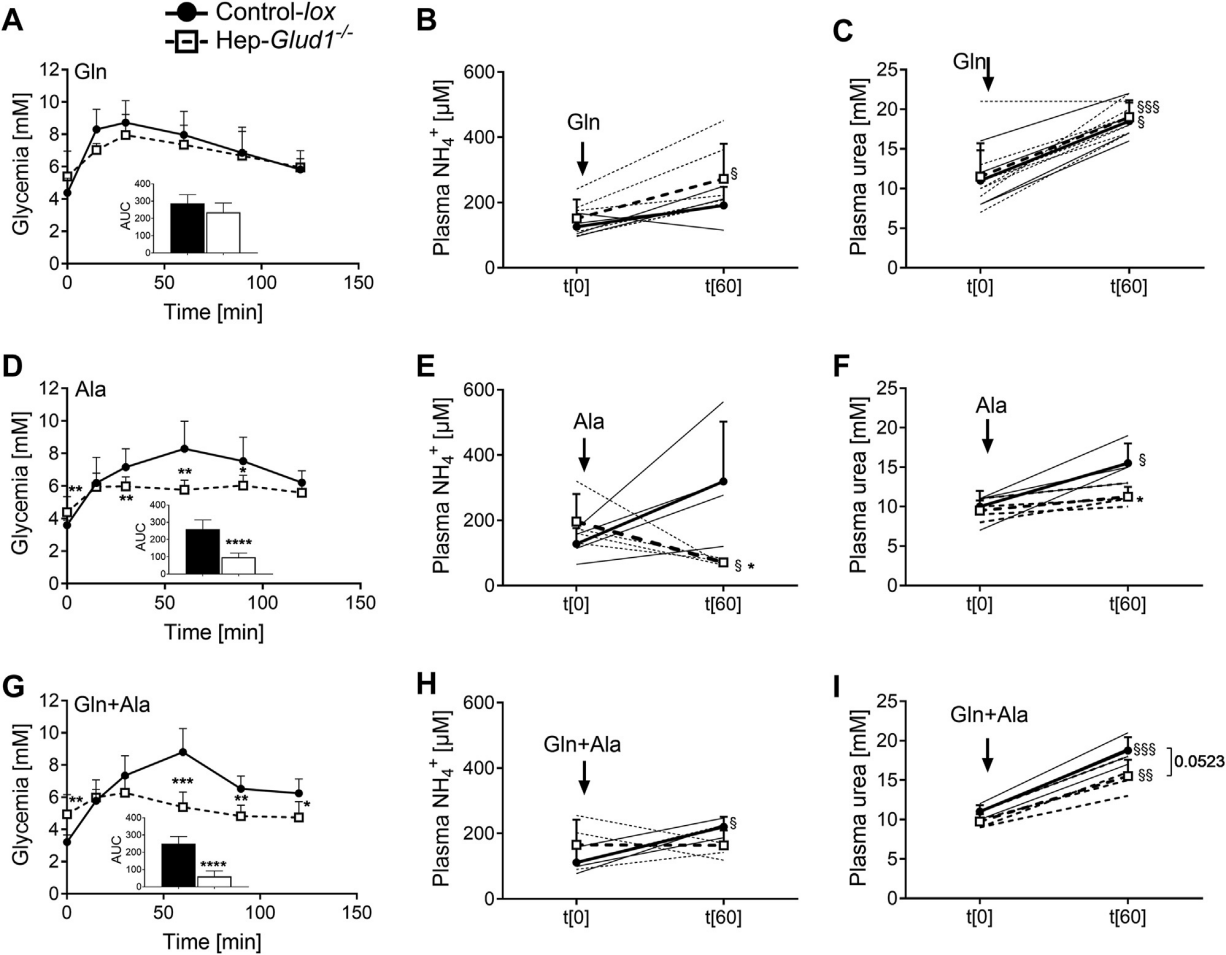
**GDH-dependent gluconeogenesis, ammonia production, and ureagenesis in mice upon amino acid challenge.** Following overnight starvation, Control-*lox* and Hep-*Glud1*^*−/−*^ mice were challenged with amino acids by i.p. injection of glutamine (*A–C*, Gln 2 g/kg, n= 4–8), alanine (*D–F*, Ala, 2 g/kg, n= 5–12), and glutamine plus alanine (*G–I*, Gln+Ala, 1 g/kg + 1 g/kg, n = 7). Glycemia (*A*, *D*, and *G*) was measured over a 120 min period with corresponding area under the curve (AUC); shown as means ± SD. Plasma ammonia (*B*, *E*, and *H*) and urea (*C*, *F*, and *I*) were measured at times 0 and 60 min following amino acid injection. Individual values (*thin lines*) and mean values ± SD (*thick lines*) are shown for ammonia and urea plasma levels. ∗*p* < 0.05, ∗∗*p* < 0.01, ∗∗∗*p* < 0.001, ∗∗∗∗*p* < 0.0001 for Control-*lox versus* Hep-*Glud1*^*−/−*^ mice; §*p* < 0.05, §§*p* < 0.01, §§§*p* < 0.001 *versus* time 0 min of the corresponding genotype. GDH, glutamate dehydrogenase.

We next tested the same substrates *in vivo* and observed similar outcomes. In control mice, administration of glutamine and alanine efficiently raised blood glucose levels and, at the same time, the circulating levels of NH_4_^+^ and urea (Fig. 2, *A–I*). In Hep-*Glud1*^*−/−*^ mice, glutamine but not alanine increased glycemia and plasma urea. These results show that, in the absence of liver GDH, gluconeogenesis and ureagenesis essentially rely on glutamine catabolism.

### Liver GDH is instrumental for the rapid adaptation of nitrogen balance to dietary protein changes

**Figure 3:**
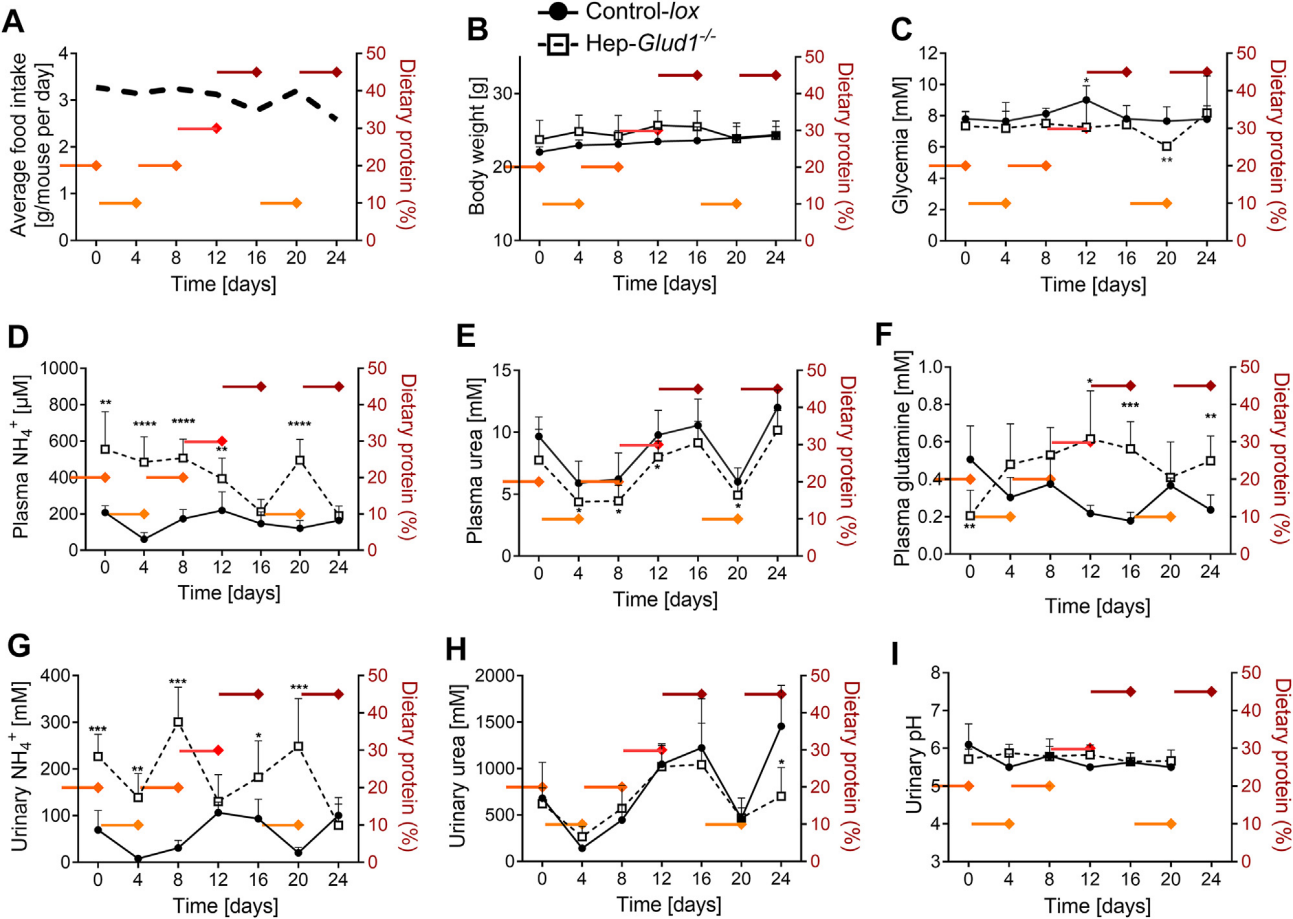
**GDH is required for ammonia homeostasis upon changes in dietary protein levels.** Control-*lox* and Hep-*Glud1*^*−/−*^ mice were fed diets with varying percentages of dietary proteins (10–20–30–45%), changed every 4 days with concomitant measurements of plasma and urine parameters (6 h after food removal). Average food intake (*A*), body weight (*B*), glycemia (*C*). Plasma concentrations (*D*) ammonia, (*E*) urea, and (*F*) glutamine. Urinary concentrations of (*G*) ammonia and (*H*) urea, and (*I*) pH. Values are means ± SD; n = 4 to 14; ∗*p* < 0.05, ∗∗*p* < 0.01, ∗∗∗*p* < 0.001, ∗∗∗∗*p* < 0.0001 for Control-*lox versus* Hep-*Glud1*^*−/−*^ mice. Food intake was measured as pellet consumption per cage of mixed genotypes. GDH, glutamate dehydrogenase.

The standard dietary protein content of laboratory mice is typically about 20% (w/w), *i.e.*, 0.6 to 1.2 g/day based on recommendations from the US National Research Council (14). Here, we sequentially challenged the mice with varying percentages of dietary proteins raging from half (10%) to twice as much (45%) the standard recommendations as follows: 10 to 20 to 30 to 45 to 10 to 45%. Diet was shifted every 4 days, without noticeable effects on average food intake and body weight in either Control-*lox* or Hep-*Glud1*^*−/−*^ mice (Fig. 3, *A* and *B*). We observed slight changes in glycemia, although remaining in a physiological range (Fig. 3*C*). Regarding nitrogen balance and ammonia detoxification, control mice could handle the high (30–45%) protein diets, limiting the elevation of plasma NH_4_^+^ levels by swiftly adapting the production of urea (Fig. 3, *D* and *E*). In the liver, the synthesis of glutamine can serve as a sink in pericentral hepatocytes for excess ammonium production if not efficiently processed into urea in the upstream periportal regions (15). Interestingly, even at 45% protein diet, control mice did not increase their plasma glutamine levels (Fig. 3*F*). This indicates that, at least on a short-term exposure, the capacity of ureagenesis was not overwhelmed, allowing efficient urinary urea excretion (Fig. 3*H*) without marked elevations of plasma and urinary NH_4_^+^ levels (Fig. 3, *D* and *G*).

In Hep-*Glud1*^*−/−*^ mice, the plasma urea levels exhibited similar adaptive patterns as the control mice, although systematically shifted below. This limited adaptative response was associated with severe hyperammonemia, elevated plasma glutamine levels, and stochastic changes in both plasma and urinary NH_4_^+^ concentrations upon fluctuations of dietary protein contents (Fig. 3, *D*, *F* and *G*). The constitutive elevation of urinary NH_4_^+^ concentrations in Hep-*Glud1*^*−/−*^ mice could also indicate higher renal glutamine catabolism for compensatory gluconeogenesis, thereby contributing to plasma glutamine clearance. Of note, none of these conditions altered urinary pH (Fig. 3*I*).

Collectively, this set of data shows that a prompt adaptation to drastic changes in dietary protein contents depends on liver GDH for an equilibrated nitrogen balance and maintenance of potentially toxic ammonium in a physiological range.

### Effects of dietary protein contents and hepatic GDH on the liver and kidney gene expression

**Figure 4:**
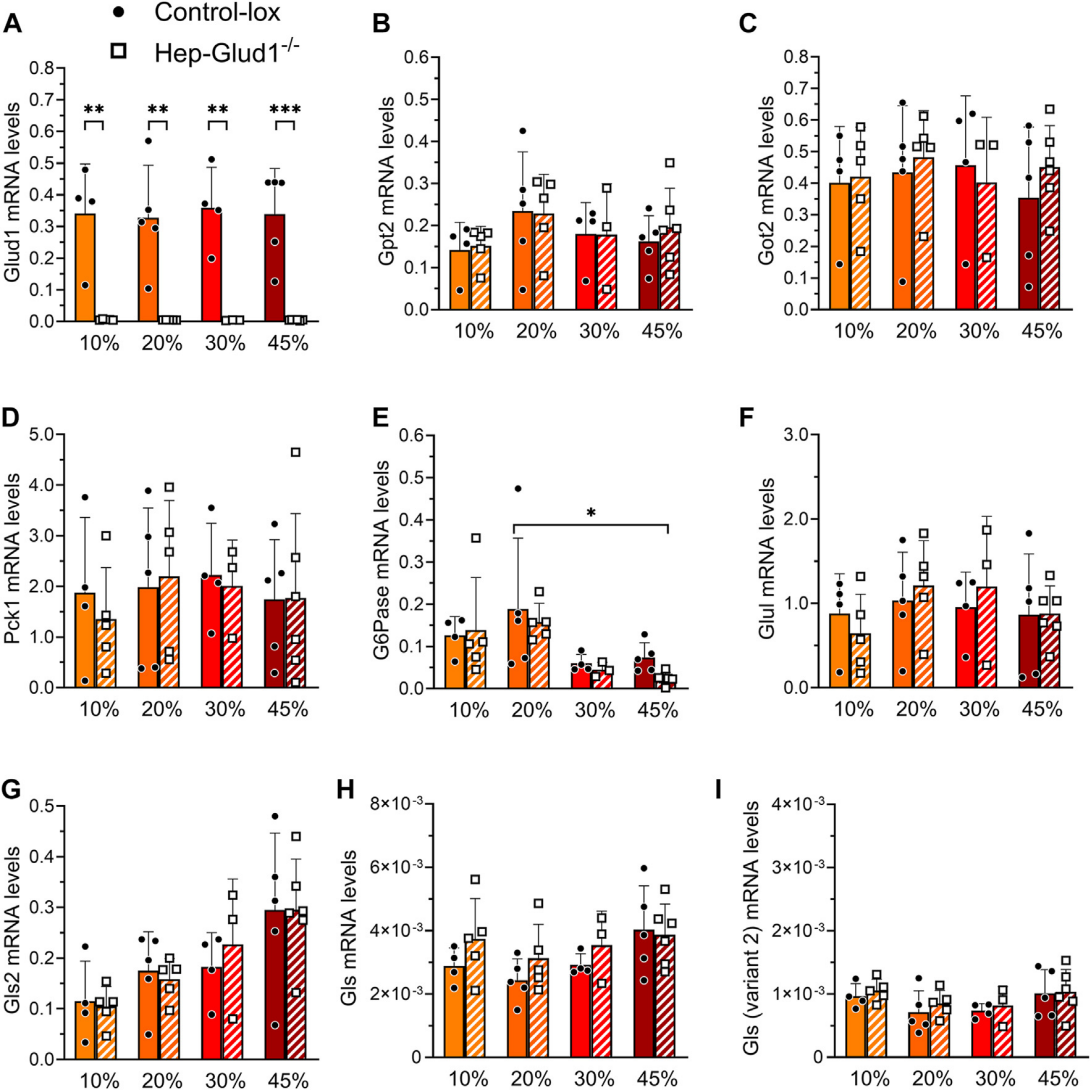
**Liver transcript levels of gluconeogenic and ammonia metabolism related genes.** Control-*lox* and Hep-*Glud1*^*−/−*^ mice were fed with 10%, 20%, 30%, and 45% protein-diet for 4 days. Transcript levels in liver cells of the glutamate dehydrogenase 1 (*Glud1*, *A*), alanine aminotransferase 2 (*Gpt2*, *B*), glutamic-oxaloacetic transaminase 2 (*Got2*, *C*), phosphoenolpyruvate carboxykinase 1 (*Pck1*, *D*), glucose-6-phosphatase (*G6Pase*, *E*), glutamine synthetase (*Glul*, *F*), liver enriched glutaminase 2 (*Gls2*, *G*), glutaminase (*Gls*, *H*; *Gls* variant 2, *I*). Results are presented as means ± SD of at least three independent experiments and expressed as mRNA levels of gene of interest normalized to cyclophilin (*Ppia*); ∗*p* < 0.05, ∗∗*p* < 0.01, ∗∗∗*p* < 0.001 for Control-*lox versus* Hep-*Glud1*^*−/−*^ mice. Pck1, phosphoenolpyruvate carboxykinase 1.

Transcript levels of genes related to ammonia and glutamine metabolisms as well as to glucose production were measured in the liver (Fig. 4) and kidney (Fig. S1) of Control-*lox* and Hep-*Glud1*^*−/−*^ mice fed for 4 days with different percentages of dietary protein. In the liver of control mice, the transcript levels of the GDH gene *Glud1* were not changed by the different diets and as expected, *Glud1* was not expressed in the liver of Hep-*Glud1*^*−/−*^ mice (Fig. 4*A*). Transaminases *Gpt2* (glutamate-pyruvate transaminase 2 or alanine aminotransferase 2) and *Got2* (glutamate-oxaloacetate transaminase 2 or AST 2) were both expressed in the liver though not changed by the high-protein diets (Fig. 4, *B* and *C*). Expression of gluconeogenic phosphoenolpyruvate carboxykinase 1 (*Pck1*) remained stable over the different diets, while glucose-6-phosphatase (*G6Pase*) was significantly repressed by the 45% protein diet (Fig. 4, *D* and *E*). Regarding glutamine metabolism, *Glul* (glutamine synthetase) mRNA levels were unchanged (Fig. 4*F*) and, among the different glutaminase variants, the liver specific *Gls2* exhibited a dietary protein dose response (Fig. 4*G*), while the two kidney specific variants were marginally expressed in the liver, without changes upon the different diets (Fig. 4, *H* and *I*). In Hep-*Glud1*^*−/−*^ mice, the gene expression profile upon changes in dietary protein contents was similar to that of control mice (Fig. 4, *B–I*). These results show that, although the high-protein diets provided extra gluconeogenic substrates, the key gene for hepatic glucose production *G6Pase* was downregulated, resulting in normal blood glucose. The liver could also adapt glutamine metabolism to the high-protein diets by upregulating the periportal glutaminase *Gls2*, with the consequence of lowering plasma glutamine levels while increasing circulating ammonium, as observed in Figure 3, *D* and *F*.

In the kidney, expression of the same set of genes was assessed. Expression of GDH-encoding *Glud1* tended to follow the increase in dietary protein contents in both control and Hep-*Glud1*^*−/−*^ mice (Fig. S1*A*). Transaminase *Gpt2* transcript levels were at low levels in the kidney as compared to the liver, while *Got2* was similarly expressed in both organs (Fig. S1, *B* and *C*). In response to the increase in protein intake, renal gluconeogenic *Pck1* exhibited a bell shape expression pattern (Fig. S1*D*), eventually correlating with glycemia in the control mice (Fig. 3*C*), while *G6Pase* did not show changes (Fig. S1*E*). Renal GS *Glul* transcript levels were unchanged, as well as the kidney specific glutaminase *Gls* variants (Fig. S1, *F–I*). A similar pattern was observed in Hep-*Glud1*^*−/−*^ mice, suggesting that kidney could handle higher glutamine catabolism without adapting *Gls* expression.

### Effects of dietary protein contents and hepatic GDH on liver and kidney protein levels

**Figure 5:**
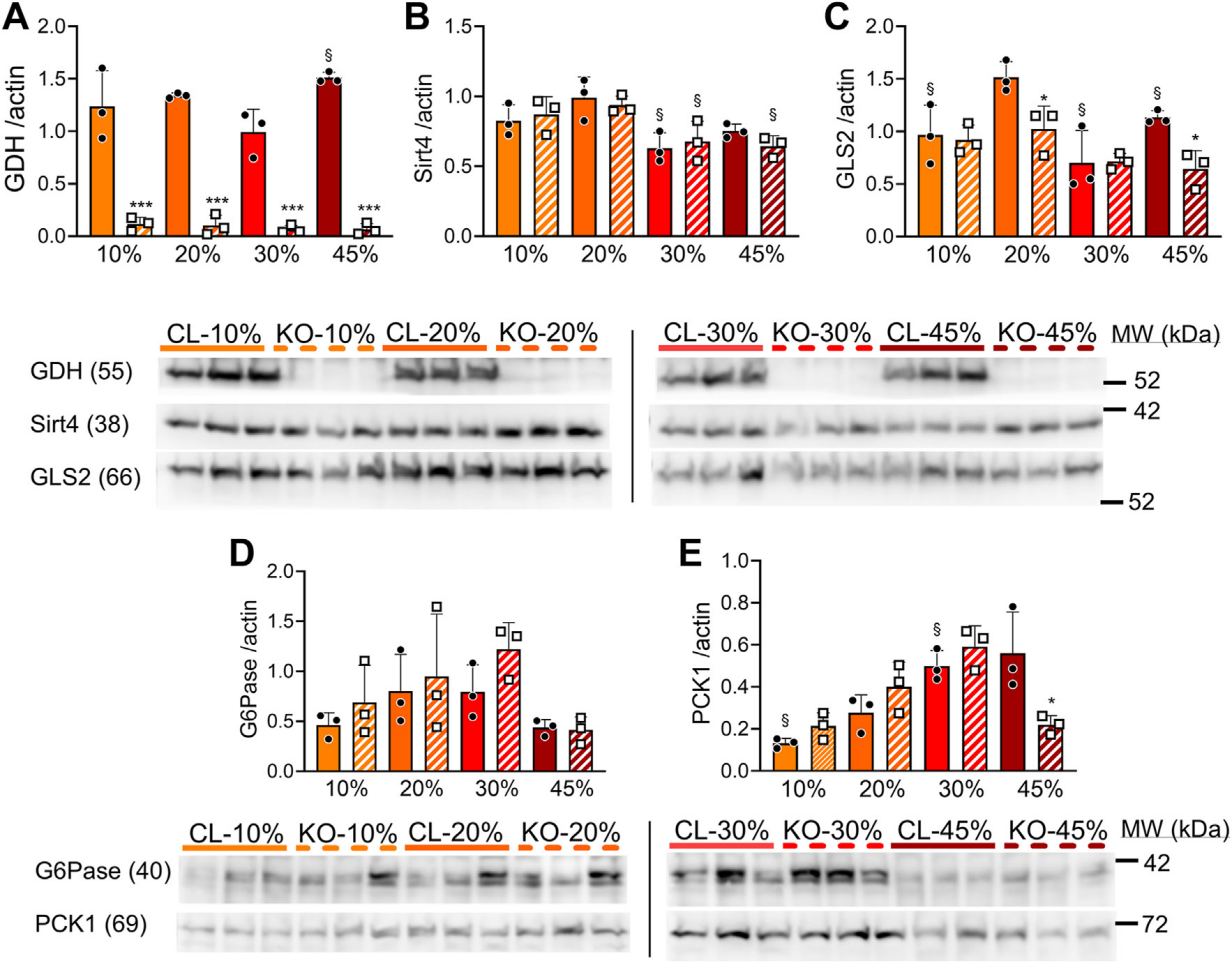
**Liver protein levels of liver gluconeogenic and GDH-dependent enzymes upon changes in dietary protein levels.** Control-*lox* (CL) and Hep-*Glud1*^*−/−*^ (KO) mice were fed diets with different percentages of dietary proteins (10–20–30–45%) for 4 days before sacrifice and tissue collection (6 h after food removal). Quantifications of immunoblotting for GDH (*A*), Sirt4 (*B*), liver-type glutaminase (*C*, GLS2), glucose-6-phosphatase (*D*, G6Pase), and PCK1 (*E*). Corresponding immunoreactive bands are shown below bar graphs. Values are mean ± SD; ∗*p* < 0.05, ∗∗*p* < 0.01, ∗∗∗*p* < 0.001 for Control-*lox versus* Hep-*Glud1*^*−/−*^ mice; §*p* < 0.05 *versus* 20% protein diet of the corresponding genotype. GDH, glutamate dehydrogenase; PCK1, phosphoenolpyruvate carboxykinase 1.

Transcript levels measured in tissues do not necessarily reflect the corresponding protein levels of the same gene, in particular upon short-term adaptations (16). Accordingly, we performed immunoblotting for top candidates identified at the mRNA level. In control mice, liver GDH was slightly upregulated by the high 45% protein diet and nearly undetectable in Hep-*Glud1*^*−/−*^ mice fed the different diets (Fig. 5*A*). The activity of GDH can be downregulated by the mitochondrial sirtuin SIRT4 through ADP-ribosylation (13). Feeding the high-protein diets (30 and 45%) reduced liver SIRT4 levels (Fig. 5*B*), potentially conferring higher GDH activity. In its catabolic function, GDH uses glutamate as a substrate that can be provided by glutaminase. The liver-type *Gls2* exhibited a dietary protein dose response pattern at the mRNA level (Fig. 4*G*), while at the protein level GLS2 was downregulated by both the high protein diet and the knockout of hepatic GDH (Fig. 5*C*). Regarding gluconeogenic enzymes, G6Pase did not exhibit significant changes (Fig. 5*D*); whereas PCK1 levels positively correlated with dietary protein contents in control mice, an adaptation that did not operate in Hep-*Glud1*^*−/−*^ mice (Fig. 5*E*).

**Figure 6:**
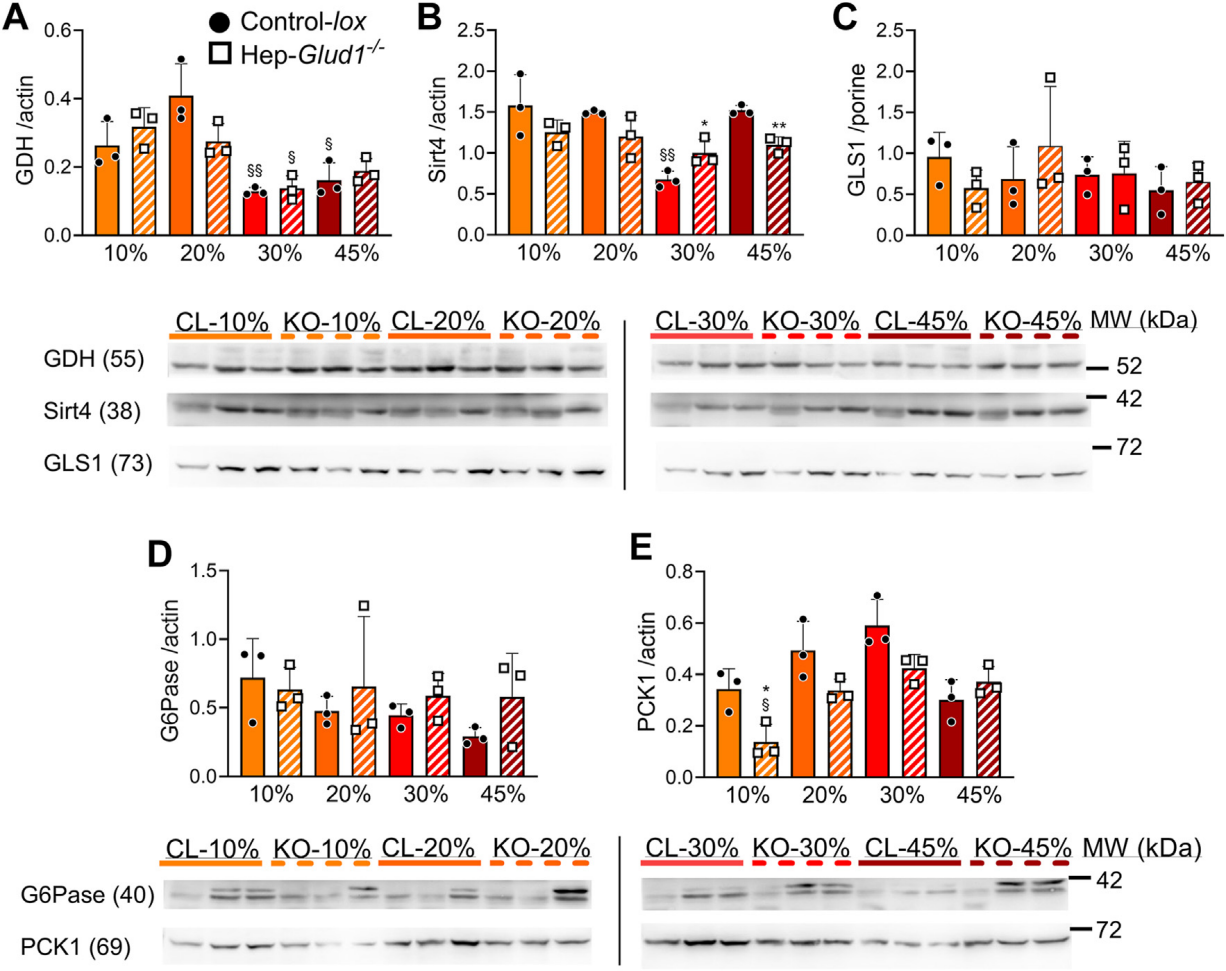
**Kidney protein levels of liver gluconeogenic and GDH-dependent enzymes upon changes in dietary protein levels.** Control-*lox* and Hep-*Glud1*^*−/−*^ mice were fed diets with different percentages of dietary proteins (10–20–30–45%) for 4 days before sacrifice and tissue collection (6 h after food removal). Quantifications of immunoblotting for GDH (*A*), Sirt4 (*B*), kidney-type glutaminase (*C*, GLS1), glucose-6-phosphatase (*D*, G6Pase), and PCK1 (*E*). Corresponding immunoreactive bands with respective loading controls (actin) are shown below bar graphs. Values are mean ± SD; ∗*p* < 0.05, ∗∗*p* < 0.01 for Control-*lox versus* Hep-*Glud1*^*−/−*^ mice; §*p* < 0.05 §§*p* < 0.01 *versus* 20% protein diet of the corresponding genotype. GDH, glutamate dehydrogenase; PCK1, phosphoenolpyruvate carboxykinase 1.

In the kidney, the high-protein diets reduced GDH protein levels, without major changes in SIRT4 (Fig. 6, *A* and *B*). Kidney-type glutaminase GLS1 did not exhibit adaptive responses to dietary protein levels, which was also the case for gluconeogenic enzymes G6Pase and PCK1 (Fig. 6, *C*–*E*). In Hep-*Glud1*^*−/−*^ mice, the kidney protein expression profile among the different dietary protein contents was similar to that of control mice (Fig. 6, *A*–*E*), showing that the lack of hepatic GDH did not impact on the renal gluconeogenic capacity and ammonia handling potential.

Overall, assessment of expression at the protein level shows the limitation of a predictive value of mRNA levels. Upon increasing dietary protein contents, the liver exhibited the major adaptive responses compared to the kidney, favoring hepatic GDH activity and the gluconeogenic capacity.

### Effects of dietary protein contents on liver and kidney GDH activity

**Figure 7:**
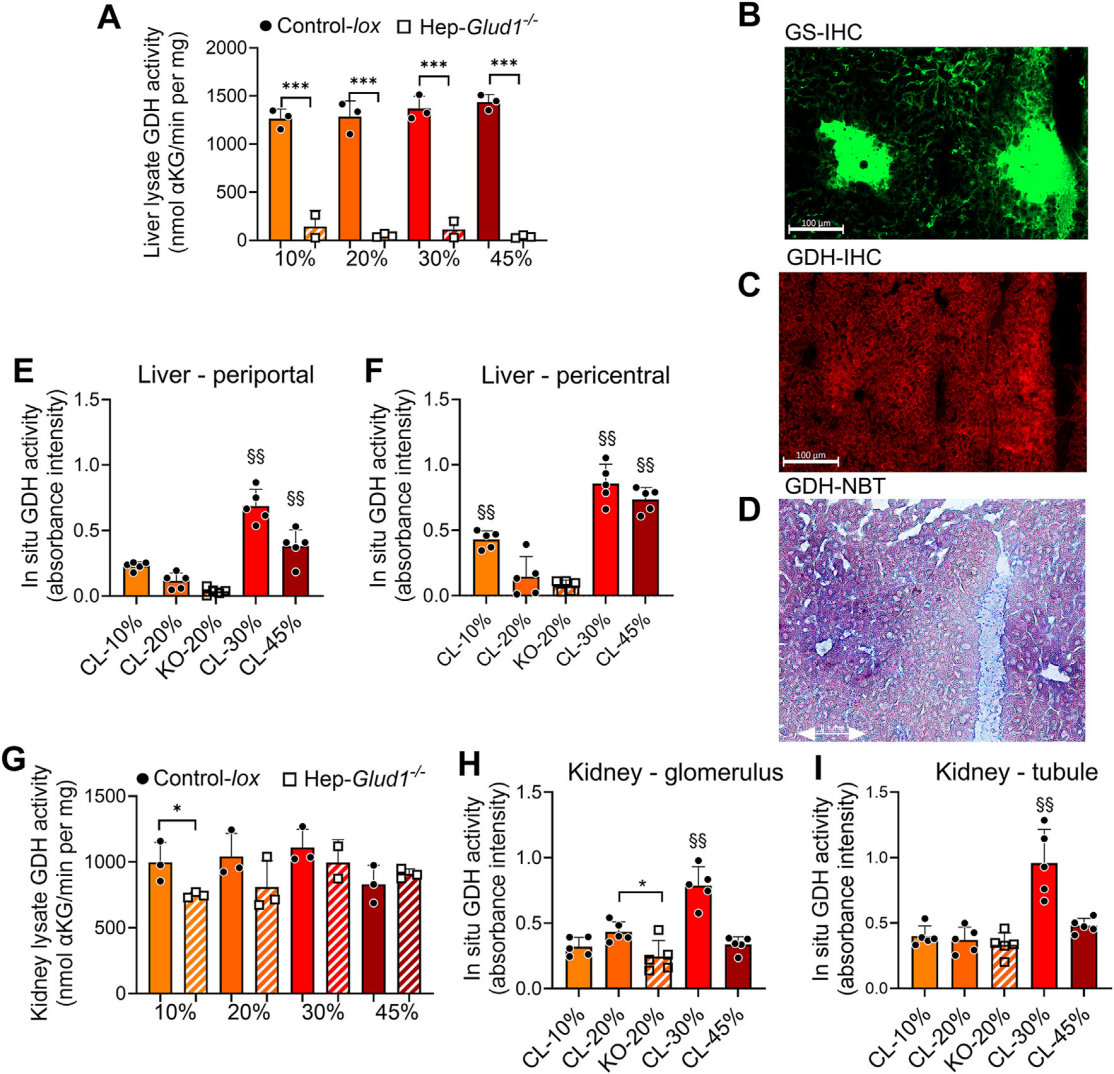
**Liver and kidney GDH enzymatic activity.** Control-*lox* (CL) and Hep-*Glud1*^*−/−*^ (KO) mice were fed diets with different percentages of dietary proteins (10–20–30–45%) for 4 days before sacrifice and tissue collection (6 h after food removal). *A*, GDH activity assessed in liver lysates. Immunohistochemistry in liver sections against (*B*) glutamine synthetase (GS) as marker for pericentral hepatocytes and (*C*) GDH (the scale bar represents 100 μm). NBT assay for GDH (*D*) representative colorimetric image on the analyzed liver section and quantification for (*E*) liver periportal region, (*F*) liver pericentral region. *G*, GDH activity assessed in kidney lysates. NBT assay and quantification for GDH on kidney sections for (*H*) glomerulus region and (*I*) tubule region. Values are expressed as means ± SD; ∗*p* < 0.05, ∗∗∗*p* < 0.01 for Control-*lox versus* Hep-*Glud1*^*−/−*^ mice; §§*p* < 0.01 *versus* 20% protein diet of the corresponding genotype. GDH, glutamate dehydrogenase; NBT, nitro blue tetrazolium.

Immunoblotting indicated potentially higher hepatic GDH activity with the high-protein diet, which was then measured in liver extracts. Although absent in Hep-*Glud1*^*−/−*^ mice, hepatic GDH enzymatic activity of control mice was unchanged by the different diets when assessed on liver lysates (Fig. 7*A*). However, homogenates miss tissue structure in general and hepatic zonation in particular, which remain preserved in cryosections (15). GS (*Glul*) immunostaining was used to reveal the pericentral regions in cryopreserved liver sections (Fig. 7*B*). Immunoreactive GDH was evenly distributed, being similarly expressed in both periportal and pericentral zones (Fig. 7*C*). We then applied *in situ* nitro blue tetrazolium (NBT) enzymatic assay to measure GDH activity directly on subsequent cryopreserved sections, revealing the strongest activity in the pericentral regions of control mice (Fig. 7*D*) and, as negative control, background signal in liver sections of Hep-*Glud1*^*−/−*^ mice (Fig. S2). Quantification of *in situ* enzymatic activity showed that the high-protein diets (30 and 45%) significantly increased GDH activity in periportal as well as pericentral zones, while the latter region exhibited the highest velocity (Fig. 7, *E* and *F*). Such *in situ* assessment of liver GDH activity reveals its zonation and an adaptive response to high-protein diets, not detected by other commonly used techniques.

Like the liver, the kidney is organized in functional units, the nephron composed of glomerulus and the tubule regions. GDH activity measured in kidney lysates did not show significant differences according to the dietary protein contents, and liver-specific GDH KO slightly reduced the renal GDH activity but only in Hep-*Glud1*^*−/−*^ mice fed with the 10% protein diet *versus* their respective controls (Fig. 7*G*). When processed *in situ* on kidney cryosections, mapping of GDH activity restricted to nephron regions revealed an increased enzymatic activity in both the glomerulus and the tubule regions when mice were fed the 30% protein diet (Fig. 7, *H* and *I*). This upregulated GDH activity did not develop in mice under the 45% protein diet, showing a bell shape effect of the dietary protein content on nephron GDH.

### Effects of dietary protein contents and hepatic GDH on the hormonal fasting response

**Figure 8:**
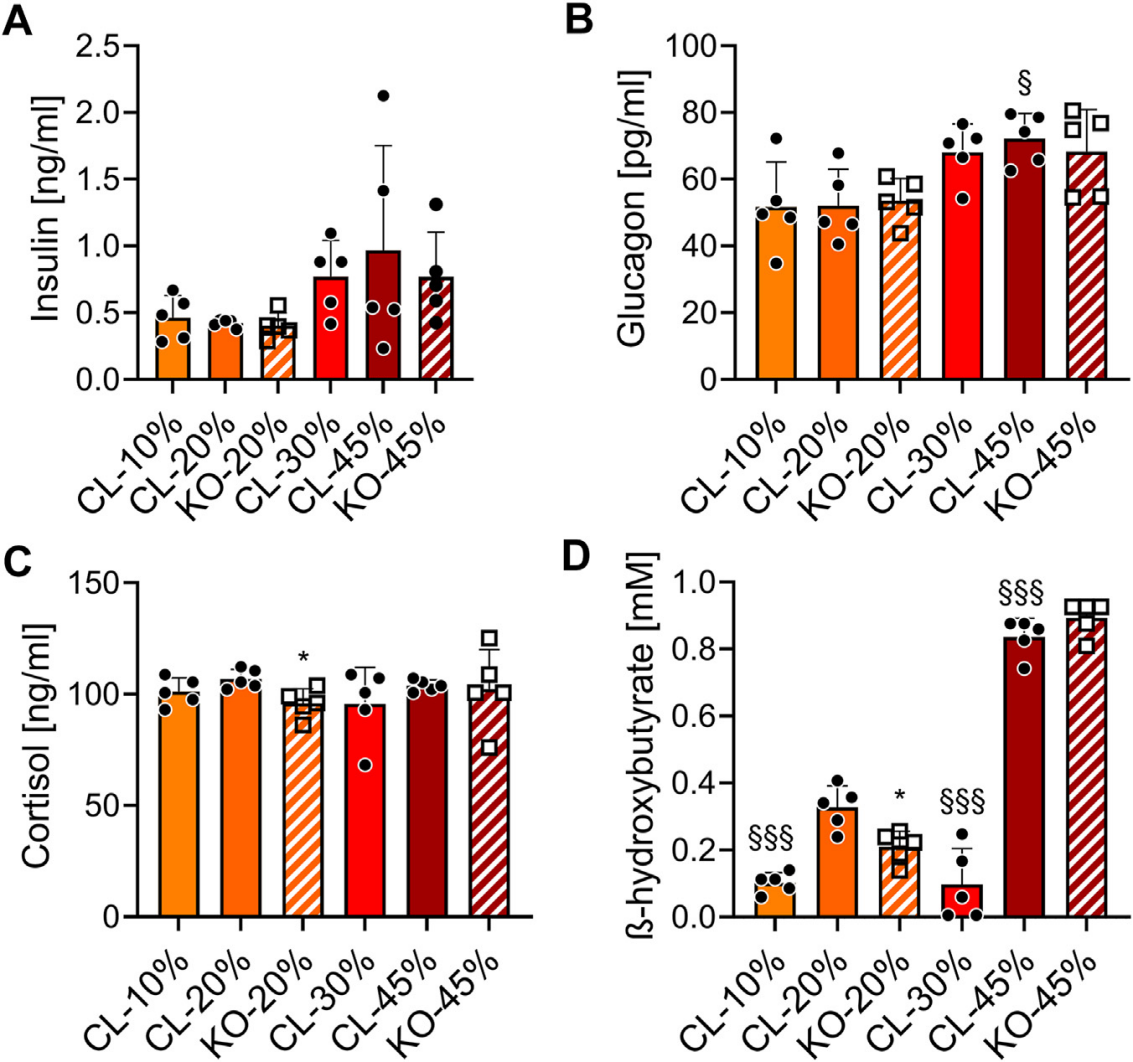
**Plasma levels of markers for energy homeostasis.** Control-*lox* (CL) and Hep-*Glud1*^*−/−*^ (KO) mice were fed diets with different percentages of dietary proteins (10–20–30–45%) for 4 days before sacrifice and tissue collection (6 h after food removal). Plasma concentrations of (*A*) insulin, (*B*) glucagon, (*C*) cortisol, and (*D*) ketone bodies. Values are expressed as means ± SD; ∗*p* < 0.05 for Control-*lox versus* Hep-*Glud1*^*−/−*^ mice; §*p* < 0.05, §§§*p* < 0.001 *versus* 20% protein diet of the corresponding genotype.

Based on the increased gluconeogenic capacity conferred by the high-protein diet, plasma concentrations of the main hormones regulating energy homeostasis were determined after a 6 h fasting period when liver glycogen stores become exhausted (10). Fasting plasma insulin levels were not influenced by the protein intake or the absence of the liver GDH (Fig. 8*A*). However, circulating glucagon was elevated by the high 45% protein diet (Fig. 8*B*), while cortisol remained at basal levels (Fig. 8*C*). Although not considered as a ketogenic diet *per se*, feeding mice for 4 days with 45% protein diet resulted in increased levels of plasma ß-hydroxybutyrate, reaching 0.836 ± 0.025 mM in a 6 h fasting state (Fig. 8*D*); while in these conditions glycemia remained in a normal range (Fig. 3*C*). Based on these results, we next focused the study on the high-protein diet and investigated the metabolic adaptations at the circadian level when switching from 20% to 45% protein diet and back.

### High protein diet exacerbates changes in fatty acid oxidation of Hep-*Glud1*^*−/−*^ mice

**Figure 9:**
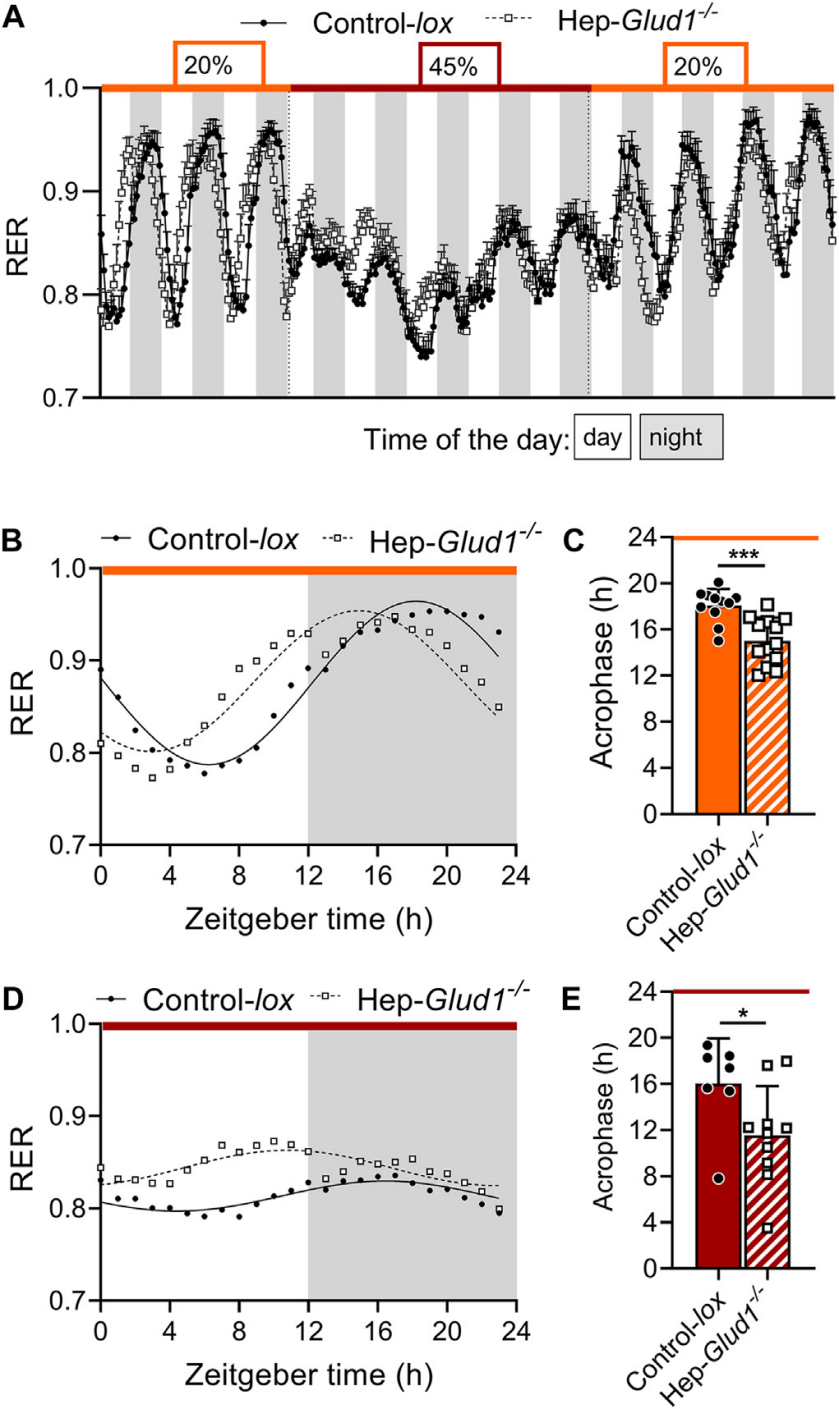
**Lack of hepatic GDH induces phase advance of the circadian rhythm of respiratory exchange ratio (RER), which is exacerbated by high-protein diet.***A*, hourly RER of Control-*lox* and Hep-*Glud1*^*−/−*^ mice fed 20% protein diet (*orange*), then switched to 45% protein diet for 5 days (*dark red*), and finally switched back to 20% protein diet and monitored for 4 days (*orange*). *B*, cosine wave and (*C*) acrophase of RER rhythm during the initial 20% protein diet. *D*, cosine wave and (*E*) acrophase of RER rhythm during the second day of 45% protein diet. For the initial 20% protein diet, the 3 days of recording were averaged. Data are shown as mean ± SD. Differences were calculated as Student’s *t* test for *C* and *E*. ∗*p* < 0.05, ∗∗∗*p* < 0.001 Control-*lox versus* Hep-*Glud1*^*−/−*^ mice. GDH, glutamate dehydrogenase; RER, respiratory exchange ratio.

In agreement with our previous observations (10), when fed a standard 20% protein diet Hep-*Glud1*^*−/−*^ mice exhibited a phase advance in the respiratory exchange ratio (RER), which is calculated by the ratio of the production of CO_2_ and the consumption of O_2_ at any given time (Fig. 9, *A–C*). RER fluctuates in between its two theoretical extreme values: 0.7 indicating utilization of lipids, mostly seen during the resting diurnal phase (fasting daytime for mice) and 1.0 for usage of carbohydrates, essentially associated with the active eating phase at night (Fig. 9*A*). When mice were switched to the high 45% protein diet, control and knockout mice displayed different adaptive responses throughout time. RER was lowered in both groups, reflecting the low carbohydrate content of the new diet. However, during the light phase of the first 2 days, Hep-*Glud1*^*−/−*^ mice had higher utilization of carbohydrates *versus* control mice, as shown by an RER closer to 1.0 (Fig. 9*A*). By the second day on the 45% protein diet, the RER of both groups showed flattened oscillatory pattern around 0.8; with a phase advance maintained for the Hep-*Glud1*^*−/−*^ mice (Fig. 9, *D* and *E*). When mice were switched back to the regular 20% protein diet the adaptation was again dynamic. Indeed, by the second day the phases of RER rhythms were synchronized and, by the fourth day of recording, all mice returned to their previous energy substrate utilization. We analyzed the rhythmicity of the RER by applying cosinor analysis (17), which underscored the phase advance of the Hep-*Glud1*^*−/−*^ animals *versus* control mice when calculating their respective acrophases (time of the day where the cosine adjusted curve of the RER reaches its maximum); Figure 9, *B–E*. This phase advance observed in the absence of liver GDH was maintained throughout the 45% protein diet period.

As an important modulator of the RER, fatty acid oxidation is calculated from the RER and the heat produced at any given time (18). During the initial phase at 20% protein diet, Hep-*Glud1*^*−/−*^ mice consumed fewer lipids at daytime compared with control animals and showed no overall circadian alternance between dark and light phases (Fig. S3). After switching to a 45% protein diet, the peak of fatty acid oxidation moved to the eating dark phase for both groups, and the overall source of energy substrate was shifted toward lipids (Fig. S3). When the 20% protein diet was restored, both groups rapidly regained carbohydrate oxidation. These results show that a high-protein diet restricts carbohydrate consumption. Consequently, the RER oscillates out of the 1.0 carbohydrate zone, resulting in a blunted pattern of circadian variations in the RER.

### The lack of liver GDH reduces voluntary activity on a high-protein diet

**Figure 10:**
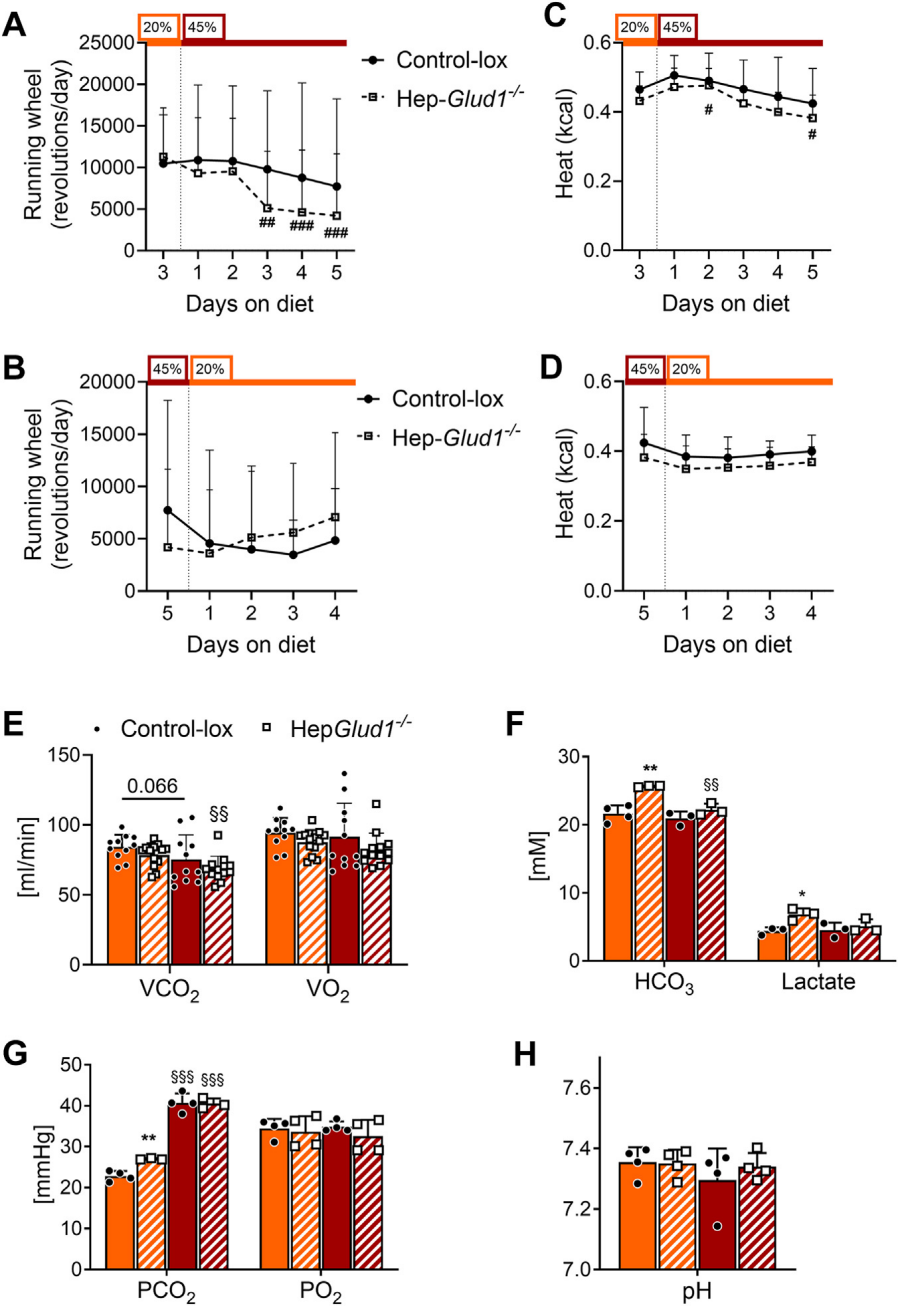
**Reduced activity and compensatory hypoventilation counteract metabolic alkalosis in Hep-*Glud1***^***−/−***^**mice.***A*–*D*, Voluntary activity (free access to running wheels; *A* and *B*) and heat production (*C* and *D*) of Control-*lox* and Hep-*Glud1*^*−/−*^ mice fed 20% protein diet (*orange*), then switched to 45% protein diet (*dark red*), and back. Mice fed with either standard (20%) or high (45%) protein diet for 4 days before blood sampling 6 h after food removal: *E*, VCO_2_ and VO_2_ measured during the 12 h of dark phase in calorimetric chambers after 4 days of adaptation; (*F*) bicarbonate and lactate blood concentrations; (*G*) blood partial pressure of CO_2_ and O_2_; (*H*) blood pH. Values are means ± SD, n= 11 to 12 (*E*) and 3 to 4 (*F* and *G*); ∗*p* < 0.05, ∗∗*p* < 0.01 Control-*lox versus* Hep-*Glud1*^*−/−*^ mice; §§ *p* < 0.01, §§§ *p* < 0.0001 20% *versus* 45% protein diet of the corresponding genotype. VCO_2_, volume of carbon dioxide produced; VO_2_, volume of oxygen consumed.

The availability and source of energy substrates may impact on physical activity and, by extension, on voluntary exercise reflecting physical performance and endurance in mice, although with substantial variations among animals (19). Voluntary activity was tested in cages equipped with running wheels to which individually housed mice had free access to. We found no difference in the running wheel pattern or distance traveled between the control and Hep-*Glud1*^*−/−*^ mice when fed a standard 20% protein diet (Fig. 10*A*). When switched to the 45% protein diet, Hep-*Glud1*^*−/−*^ mice significantly reduced their spontaneous activity after a couple of days, while control animals maintained a similar level of activity. After the 20% protein diet was reestablished, Hep-*Glud1*^*−/−*^ mice partially restored their voluntary activity at levels approaching the basal condition (Fig. 10*B*). At baseline, energy expenditure was not different between the control and Hep-*Glud1*^*−/−*^ mice (Fig. 10*C*). When they switched to the 45% protein diet this parameter was transiently increased and after the third day on high protein Hep-*Glud1*^*−/−*^ mice significantly reduced their energy expenditure (Fig. 10*C*). Once back on the 20% protein diet, control and Hep-*Glud1*^*−/−*^ mice stabilized heat production to baseline (Fig. 10*D*).

Indirect calorimetry confirmed reduced energy expenditure in Hep-*Glud1*^*−/−*^ mice fed the 45% protein diet (Fig. 10*E*). At this stage, it is unclear if the lower voluntary activity recorded in Hep-*Glud1*^*−/−*^ mice on a high-protein diet is a cause or the consequence of reduced energy expenditure compared to control mice. Hep-*Glud1*^*−/−*^ mice constitutively excrete more concentrated urinary ammonia *versus* control animals (Fig. 3*G*). Moreover, a high-protein diet has diuretic effects and is associated with an increase in water intake (20), which was confirmed in the present study (Fig. S4). Consequently, an elevated loss of NH_4_^+^ is expected to promote metabolic alkalosis inducing an increase in plasma HCO_3_^−^, which was indeed witnessed in Hep-*Glud1*^*−/−*^ mice (Fig. 10*F*). This in turn should promote compensatory hypoventilation in order to increase the partial pressure of carbon dioxide. PCO_2_ was higher in Hep-*Glud1*^*−/−*^ mice on a standard 20% protein diet and was robustly elevated in both control and liver GDH null mice on the 45% protein diet (Fig. 10*G*). The net anticipated effect of this respiratory adaptive compensatory response is to maintain the HCO_3_^−^/PCO_2_ ratio, eventually preserving blood pH. This homeostatic mechanism was successful in all experimental groups resulting in physiological pH (Fig. 10*H*).

## Discussion

During fasting, glucose supply from the digestive track becomes progressively limiting. The response of the organism is to preserve glucose for obligatory glucose-dependent organs with simultaneous glucose production by the liver through glycogenolysis, followed by gluconeogenesis. For the latter, glutamine, alanine, lactate, and glycerol may be used. As gluconeogenic pathway progressively dominates the overall glucose production, the accompanying release of gluconeogenic amino acids from muscles is promoted. Once deaminated in the liver, they provide net supply into carbon pool *via* anaplerosis. GDH fulfills here essential role by bridging amino acid and carbohydrate metabolism, fueling the tricarboxylic acid cycle and providing gluconeogenic substrates. This cycling of nutrients between skeletal muscles and liver is vital for glucose homeostasis but must be tightly controlled to prevent excessive erosion of protein mass.

Sustained provision of amino acids to the liver can also be favored by protein-rich diets, such as the Paleolithic diet (containing about 30% of proteins) used nowadays for weight loss maintenance and prevention of type 2 diabetes (21). In such nonfasting situation, the concentration of amino acids, including the GDH allosteric activator leucine, increases and can activate liver GDH to accommodate surplus amino acids for energy homeostasis. In the present study, the purpose of the incremental protein intake was to study the role of liver GDH in the rapid adaptation of hepatic metabolism to fluctuations in dietary amino acids and nitrogen load. In order to delineate the metabolic responses to changes in protein intake, control and Hep-*Glud1*^*−/−*^ mice were fed chow diets with a wide coverage of protein contents: suboptimal 10%, standard 20%, over optimal 30%, and high 45% protein diets; switched every 4 days. In a study on rats maintained on a high-protein diet over 40 days, steady state of enzymatic and hormonal responses was achieved after 24 days (22). Accordingly, the changes we observed in the current study are arbitrarily considered as short-term adaptive responses. The ornithine cycle, and its product urea, is a primary system to respond to varying protein contents in a diet. It has been demonstrated that adaptations to fluctuating levels of dietary protein load is associated with changes in urea cycle enzyme activities (22) and differences in liver amino acid contents, mainly glutamate (which is the source of one ammonium through the action of GDH) and aspartate (which provides the second nitrogen for urea synthesis) (23). In agreement with previous observations (24), we observed linear relationship between protein intake and plasma urea, as well as urea excretion into urine.

Ammonium is constantly produced in mammals as a metabolic waste from amino acid catabolism and is resolved in the liver where two disposal systems are operating: the ornithine cycle and GS which convert free ammonium to urea and glutamine, respectively. GDH fulfills a specific and mandatory function in each of these pathways: as the producer of ammonium for the urea cycle and of glutamate for GS. In mice fed a standard diet, deletion of liver GDH or liver GS causes a mild systemic rise in ammonium levels due to reduced detoxification into urea or glutamine, respectively (7, 10). Here, we investigated the changes in blood-acid base homeostasis in different dietary conditions impacting nitrogen metabolism of Hep-*Glud1*^*−/−*^ mice. GDH catalyzes a reversible reaction which might serve as a sink for ammonium in the liver, catalyzing glutamate formation. On a standard 20% protein diet, Hep-*Glud1*^*−/−*^ mice displayed hyperammonemia accompanied by a rise in plasma bicarbonate levels and CO_2_ partial pressure. The hepatocyte-specific deletion of GDH resulting in hyperammonemia might be the primary event in the liver, accompanied by an adaptive response in the kidney and lungs, *i.e.*, increased bicarbonate and CO_2_ partial pressure.

Surprisingly, consumption of the 45% protein diet by Hep-*Glud1*^*−/−*^ mice fully reversed the hyperammonemia observed in KO mice fed the 20% protein diet. Plasma ammonium levels dropped to control values, which was paralleled by a decrease in blood bicarbonate and elevated plasma glutamine concentrations. High-protein diet induced changes in nitrogen pool distribution in Hep-*Glud1*^*−/−*^ mice resulting in increase of glutamine levels at 30% and 45% protein diets, potentially contributed by higher GS activity in perivenous hepatocytes or lower glutamine utilization in the kidneys. Partial pressure of CO_2_ increased with elevated protein intake to similar extent in both control and Hep-*Glud1*^*−/−*^ mice. Arterial CO_2_ partial pressure reflects aerobic energy metabolism and is modified according to different macronutrients metabolism. Blood pH values remained remarkably stable regardless of ammonium levels or protein intake in Hep-*Glud1*^*−/−*^ and control mice. Plasma concentration of H^+^ is among the most tightly regulated variables in physiology since structure and function of proteins and macromolecular complexes are critically dependent upon hydrogen concentration, notably the affinity of hemoglobin to oxygen (25). Interestingly, mice lacking liver GDH spontaneously lowered physical exercise when fed a high-protein diet. These observations indicate that the reduced spontaneous activity of Hep-*Glud1*^*−/−*^ mice might be driven by constitutive higher NH_4_^+^ urinary excretion, then impacting on the behavior of the mice and ultimately preserving blood pH. This raises interesting questions regarding fatigue mechanisms upon prolonged and sustained physical activity associated with sarcopenia coupled with gluconeogenesis releasing amino acid-derived NH_4_^+^.

In conclusion, the present study demonstrates the pivotal role played by hepatic GDH in the rapid adaptation to fluctuating provision of amino acids and their disposal by the liver. Thanks to functional GDH, clearance of ammonia overload can be achieved in a safe way primarily through ureagenesis, which is impaired in the absence of liver GDH. The latter scenario not only results in hyperammonemia but also alters gluconeogenesis, energy partitioning and ultimately voluntary movements. In disease states such as hepatic encephalopathy, in which hyperammonemia plays a critical role (26, 27), pharmaceutical regulation of GDH activity by allosteric modulators (28) could potentially drive excessive ammonia into safe urea pathway. In the perspective of clinical outcomes, the challenge would be to target GDH specifically in the liver.

## Experimental procedures

### Animals

The *in vivo* deletion of GDH in hepatocytes was prompted by tamoxifen treatment of *Glud1*^*lox/lox*^ mice (*Glud1*^*tm1.1Pma*^, MGI:3835667 (29)) carrying the *Alb-CreERT2* construct (30) giving rise to liver-specific GDH-KO mice, namely Hep-*Glud1*^*−/−*^ mice characterized previously (10). The *in vivo* recombination was induced at 8 weeks of age by subcutaneous implantation of tamoxifen pellets (Tamoxifen free base, 25 mg/pellet, 21-days release, E-361; Innovative Research of America). Implantation was conducted in male *Glud1*^*lox/lox*^ mice carrying the Albumin-Cre-ER^T2^ construct (Hep-*Glud1*^*−/−*^) as well as in control floxed *Glud1*^*lox/lox*^ (Control-*lox*). The latter group was composed of littermates in order to optimize standardization of the genetic background between the two groups. Mice were maintained in our certified animal facility (12-h × 12-h light/dark cycle with 7 AM on and 7 PM off) according to procedures approved by the animal care and experimentation authorities of the Canton of Geneva (GE/166/16). Food and water were provided *ad libitum* during the duration of the experiment unless otherwise specified.

### *In vivo* experiments

Animals were maintained on SAFE-150 diet (Safe) until the age of 12 weeks, *i.e*., 4 weeks after successful tamoxifen-mediated recombination induced at 8 weeks of age. Then, both Control-*lox* and Hep-*Glud1*^*−/−*^ mice were put for sequential periods of 4 days on diets with varying protein contents: 10% protein content (D11080501; Research diets), 20% protein content (SAFE-150), 30% protein content (D10062202i; Research diets), and 45% protein content (E15209: ssniff). The food intake was assessed by weighting the remaining pelleted chow every 4 days. Where indicated, calorimetric parameters (O_2_ consumption and CO_2_ production), food and water intake, and locomotor activity were determined using a TSE PhenoMaster system (TSE Systems).

Alanine and glutamine challenges were performed after an overnight fast by i.p. injection of L-alanine (2 g/kg; Sigma-Aldrich) or L-glutamine (2 g/kg; Sigma-Aldrich) or both L-alanine (1 g/kg) and L-glutamine (1 g/kg). After amino-acid administration, blood samples were taken at the indicated time intervals (0–120 min) from the tail vain for glucose measurements or from submandibular vein at time 0 and 60 min for ammonia and urea measurements. Blood glucose levels were measured using Accu-Check Aviva glucometer (Roche Diagnostics). The total area under the curve of glycemia was calculated using the GraphPad Prism 7 software (https://www.graphpad.com). Plasma ammonia and urea were measured within 20 min after blood collection in the Laboratoire de chimie clinique of the Geneva University Hospitals. We used the epoc Blood Analysis System (Siemens Healthcare) to measure the following parameters: bicarbonate, lactate, partial pressure of O_2_ and CO_2_, and blood pH.

Upon sacrifice, blood and tissue parameters were determined on samples collected from animals fasted for 6 h (7.30 AM to 1.30 PM). Ammonia and urea from plasma and urine were measured as mentioned above. Glutamine and ß-hydroxybutyrate were determined on plasma kept at −80 °C up to 2 weeks using commercial kits (ab197011 from Abcam and K632–100 from Biovision, respectively). ELISA kits used to assess plasma levels of insulin and glucagon were from Crystal Chem and from Enzo for cortisol.

### Isolation and metabolic challenges on culture of mouse hepatocytes

Isolation and culture of primary mouse hepatocytes were performed as detailed previously (10). Briefly, hepatic portal vein of anesthetized mice was cannulated and inferior vena cava cut in order to successfully perfuse the liver with calcium-free perfusion buffer (PB) solution (142 mM NaCl, 6.6 mM KCl, 9.6 mM Hepes, and 6 mM NaOH) at 37 °C for 10 min. Then, calcium-free PB was replaced for dissociation buffer (PB with 4.76 mM CaCl_2_) plus Liberase (Roche) at 37 °C for 5 min. The liver was carefully removed and transferred to petri dishes filled with preservation buffer (PB solution with 0.01 g/ml bovine serum albumin). Glisson capsule was disrupted to release the cells and to separate them from undigested tissue through a 70 μm mesh nylon filter before multiple step purification (10) and finally resuspension in culture medium (Williams E medium, 5% fetal calf serum, 10^−9^M insulin, 10^−6^ M dexamethasone, Pen/Strep, and 1% glutamax). Hepatocytes were then seeded at 50,000 cells/cm^2^ on collagen-coated plates.

For metabolic challenges culture medium was replaced by Dulbecco's modified Eagle's medium 5030 without glucose, glutamine, and pyruvate (Sigma-Aldrich) for glycogen depletion. After such 6 h starvation period, cells were challenged for 1 h (37 °C) with 5 mM alanine and/or 5 mM glutamine in Krebs-Ringer bicarbonate Hepes buffer (140 mM NaCl, 3.6 mM KCl, 0.5 mM NaH_2_PO_4_, 0.5 mM MgSO_4_, 2 mM NaHCO_3_, 1.5 mM CaCl_2_, and 10 mM Hepes) or Krebs-Ringer bicarbonate Hepes without amino acids for basal conditions. Supernatant was collected and used for product analysis. NH_3_ was measured within 20 min after collection by the Laboratoire de chimie clinique. Urea and glucose concentrations were determined using commercial kits (K375-100 and K606-100, respectively; Biovision).

### RNA analyses

Total RNA from different organs was extracted using peqGOLD Trifast (Peqlab)/chloroform. RNA concentration was determined using the NanoDrop device (Thermo Fisher Scientific), and its integrity was assessed by migration onto a 2%-agarose gel. Samples were treated with DNase I (DNA removal kit, Thermo Fisher Scientific) and reverse transcription was performed using SuperScript II reverse transcriptase; complementary DNA was stored at −20 °C.

Then, real-time quantitative reverse transcription-PCR was performed on complementary DNA samples using the StepOnePlus real-time PCR device (Applied Biosystems). Target genes were amplified using SybrGreen technology and the ΔΔCT method. Cyclophilin (*Ppia*) was used as reference. Primers are listed in the supplementary material (Table S1).

### Enzymatic activity

GDH activity was measured from tissue extract of the liver and kidney lysed in 20 mM Tris–HCl (pH 8.0) with 2 mM cyclohexanediaminetetraacetic acid and 0.2% Tween-20. GDH activity was detected in the oxidative deamination direction in 50 mM Tris–HCl buffer (pH 9.5, 2.6 mM EDTA, 1.5 mM NAD, and 1 mM ADP). The reaction was initiated by the addition of 4 mM glutamate, and NADH autofluorescence was recorded over a 20 min period at 37 °C on a Fluostar Optima (BMG Labtech). GDH enzymatic activity was calculated as initial velocity compared to standard curve obtained from the activity of purified liver GDH as detailed previously (31).

*In situ* GDH activity has been assessed on liver and kidney cryosections by quantitative enzyme histochemistry using the NBT assay described previously (32). Practically, when mice were sacrificed, parts of their liver or kidney were embedded in optimal cutting temperature compound matrix (CellPath) and stored at −80 °C before analysis. Then, cryosections (7 μm) were prepared and allowed to reach 37 °C in a dark humid chamber before addition of PBS buffer (pH 8.0, 0.1 M KH_2_PO_4_, and 0.1 M Na_2_HPO_4_) with 10% of polyvinyl alcohol (Alfa Aesar), 0.32 mM phenazine methosulfate (Sigma-Aldrich), 5 mM NBT (prepared in 1:1 ethanol:dimethylformamide), 1.0 mM ADP (Sigma-Aldrich), 1.5 mM NAD^+^ (AppliChem), and 4.0 mM glutamate as substrate. After 30 min, reaction was stopped by washing the preparation with PBS at 60 °C and then with Milli-Q water. Images were acquired under brightfield light on a Zeiss Axio Scan Z1 scanner using a 5× objective. For quantification of *in situ* activity, images were analyzed using QuPath software (https://qupath.github.io/) and regions of interest were determined based on GS positive *versus* negative zonation according to the immunohistochemistry performed on subsequent sections from the same sample. For each section, absorbance values were measured on five different zones.

### Immunodetection on Western blotting and immunohistochemistry

For Western blotting, protein extracts (30 μg) were prepared from tissues homogenized in radioimmunoprecipitation assay buffer and separated on bis-acrylamide 10% gel, then probed with specific antibodies (see detailed list, suppliers, and dilutions in Table S2). Bands were revealed by horseradish peroxidase assay and quantification was performed using the Syngene PXi gel imaging system (Thermo Fischer Scientific).

For immunohistochemistry, liver cryosections described above were fixed in cold acetone and then blocked in PBS with 0.4% Triton X-100 before overnight incubation at 4 °C with primary antibodies against GDH or GS (see suppliers and dilutions as in Table S2). On the next day, sections were washed with PBS and incubated with secondary antibodies at room temperature for 2 h; then washed again in PBS before image acquisition on Leica LSM800 Airyscan microscope (Leica).

### Statistical analysis

Data are presented as means ± SD. Statistical analyses were performed using GraphPad Prism 9.5 software. For more than two groups of data sets, one-way ANOVA analysis was used followed by Tukey multiple comparison test and Student’s *t* test when only two groups of data were compared. The difference was considered significant when *p* < 0.05.

## Data availability

All data generated during these studies are included in the text, figures, and tables of this article and electronic supplementary material. Source data or materials will be supplied by the corresponding authors with reasonable request.

## Supporting information

This article contains supporting information.

## Conflict of interest

The other authors declare that they have no conflicts of interest with the contents of this article.
