# Supplementary material for: Dietary protein load affects the energy and nitrogen balance requiring liver glutamate dehydrogenase to maintain physical activity
Source: J Biol Chem. 2024 Jun 13;300(7):107473. doi: 10.1016/j.jbc.2024.107473 (PMC11301064; doi:10.1016/j.jbc.2024.107473)
Supplement: Supplementary Table S1 and Figures S1–S4 [file mmc1.pdf]

Supporting Information

Luczkowska et al.

Supplementary Table S1: primers used for quantitative RT-PCR analysis.

**Primer symbol**	**Gene ID (NBCI)**	**Aliases**	**Full name**	**Sequence**
Glud1	#14661	*Gdh*	glutamate dehydrogenase 1	Fwd:5’-TGGGCCTGCACTCTATGAGAT-3’<br>Rev:5’-CTCCCATCAGACTCTCCAACAC-3’
Gpt2	#108682	*Alat2*	*alanine aminotransferase 2*	Fwd:5’-TTGCCTGGGAAGAGAAGCTTT-3’<br>Rev:5’-GCAGTCTGGAGAGTACACGTTGTC-3’
Got2	#14719	*Asat*	*glutamatic-oxaloacetic transaminase 2*	Fwd:5’-CCAATCGTATGCCAAGAACATG-3’<br>Rev:5’-TGGCTTCTTCTGCATCTTTGC-3’
Glul	#14645	*Gs*	*glutamine synthetase*	Fwd:5’-CAGGCTGCCATACCAACTTCA-3’<br>Rev:5’-TGGCCTCCTCAATGCACTTC-3’
Pck1	#18534	*Pepck1*	phosphoenolpyruvate carboxykinase 1	Fwd:5’-TGGAAGGTCGAATGTGTGGG-3’<br>Rev:5’-CAGTAAACACCCCCATCGCT-3’
G6Pase	#14377	*G6Pc*	glucose-6-phosphatase	Fwd:5’-GTGGCAGTGGTCGGAGACT-3’<br>Rev:5’-ACGGGCGTTGTCCAAAC-3’
Gls	#14660	*Gls1*	glutaminase	Fwd:5’-CTGTAGATGGGCAAAGGCATTC-3’<br>Rev:5’-TCAGTTCCCAGGTCATTAACAGC-3’
Gls (variant 2)	#14660	*Gls1 (variant2)*	glutaminase	Fwd:5’-CTGAGTCAAGTGACGACACCTCT-3’<br>Rev:5’-GCTAGAGCCCATCTCTAGCTCCT-3’
Gls2	#216456	*Gls2*	*glutaminase 2 (liver, mitochondrial)*	Fwd:5’-ACAAGACCGTGGTGAACCTGC-3’<br>Rev:5’-GGGCTGTGCGGCAATCATAGT-3’
Ppia	#268373		*peptidylprolyl isomerase A; cyclophilin A*	Fwd:5’-ATCTGCACTGCCAAGACTGA-3’<br>Rev:5’-TCTTGCTGGTCTTGCCATTC-3’

Supplementary Table S2: antibodies used for immunodetection on western blotting (WB) and immunohistochemistry (IHC).

Target	Name	Supplier	Reference	Dilution
Glutamate dehydrogenase (GDH)	Rabbit anti-GDH	Rockland	100-4158	1:2000 (WB)
Glutamate dehydrogenase (GDH)	Rabbit anti-GDH	Cell Signaling	D9F7P	1:500 (IHC)
Sirtuin 4 (Sirt4)	Goat anti-SIRT4	Abcam	ab10140	1:1000 (WB)
Glutaminase 2 (GLS2)	Rabbit anti-GLS2	Abcam	ab113509	1:2000 (WB)
Glutaminase 1 (GLS1)	Rabbit anti-GLS1-HRP	Abcam	ab200408	1:2500 (WB)
Glutamine synthase (GS)	Mouse anti-GS	Millipore	MAB302	1:500 (IHC)
Glucose-6-phosphatase (G6pase)	Rabbit anti-G6Pase	University of Lyon	Dr. G. Mithieux	1:5000 (WB)
Phosphoenolpyruvate carboxykinase 1 (PCK1)	Rabbit anti-PCK1	Abcam	ab70358	1:2500 (WB)
Actin	Mouse anti-Actin	Millipore	MAB1501	1:5000 (WB)
Porin	Rabbit anti-Porin	Abcam	ab34726	1:2000 (WB)
Secondary	Anti-rabbit HRP	Sigma	A8275	1:4000 (WB)
Secondary	Anti-mouse HRP	Sigma	A5278	1:2500 (WB)
Secondary	Anti-goat HRP	Santa Cruz	sc-2056	1:3000 (WB)

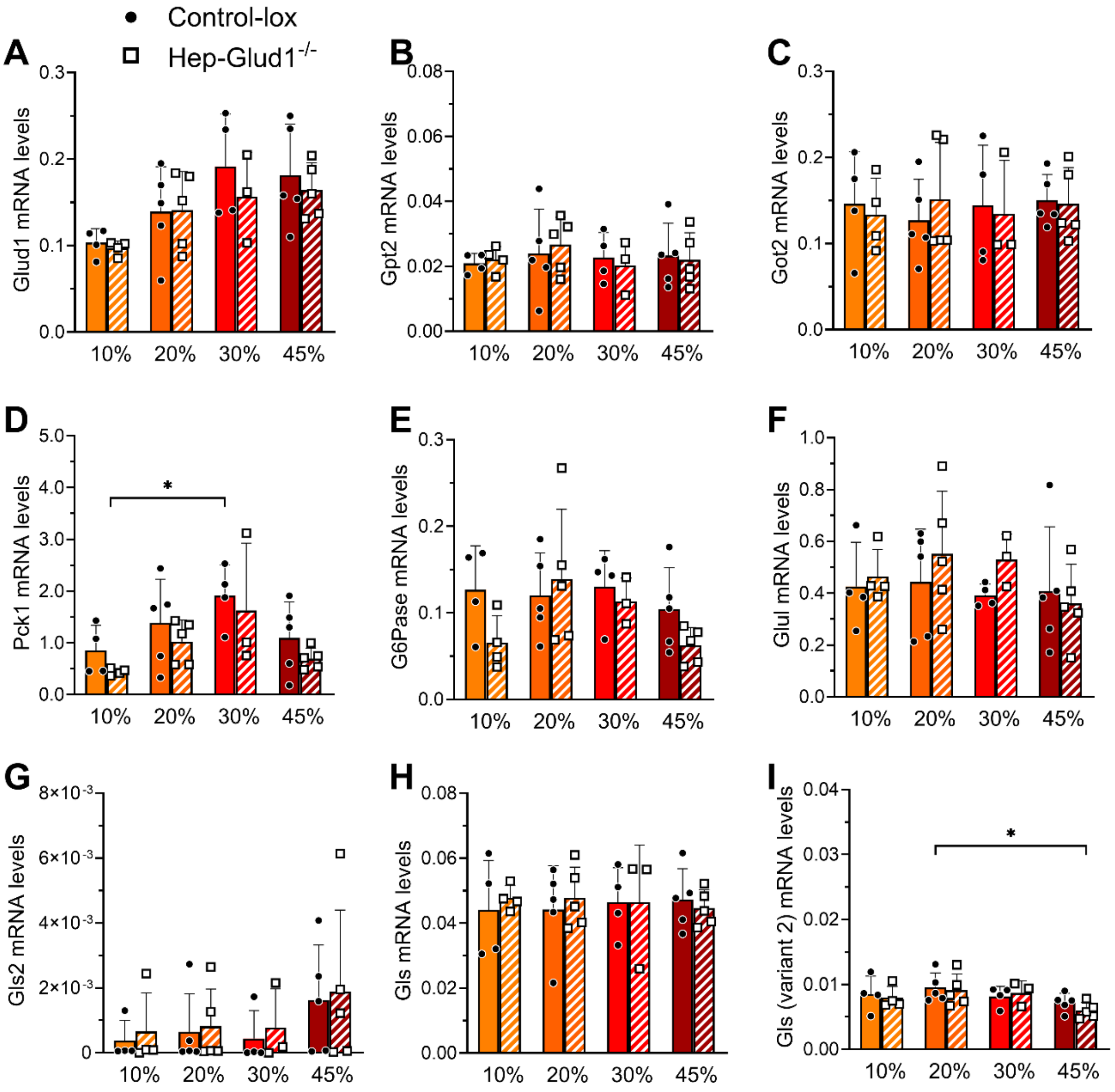

Supplementary Figure S1. Kidney transcript levels of gluconeogenic and ammonia metabolism related genes. Control-*lox* and Hep-*Glud1*$^{-/-}$ mice were fed with 10%, 20%, 30% and 45% protein-diet for 4 days. Transcript levels in renal cells of the glutamate dehydrogenase 1 (*Glud1*, **A**), alanine aminotransferase 2 (*Gpt2*, **B**), glutamic-oxaloacetic transaminase 2 (*Got2*, **C**), phosphoenolpyruvate carboxykinase 1 (*Pck1*, **D**), glucose-6-phosphatase (*G6Pase*, **E**), glutamine synthetase (*Glul*, **F**), liver enriched glutaminase 2 (*Gls2*, **G**), glutaminase (*Gls*, **H**; *Gls* variant 2, **I**). Results are presented as means ± SD of at least 3 independent experiments and expressed as mRNA levels of gene of interest normalized to cyclophilin (*Ppia*); *p<0.05, **p<0.01, ***p<0.001 for Control-lox versus Hep-*Glud1*$^{-/-}$ mice.

A **B**

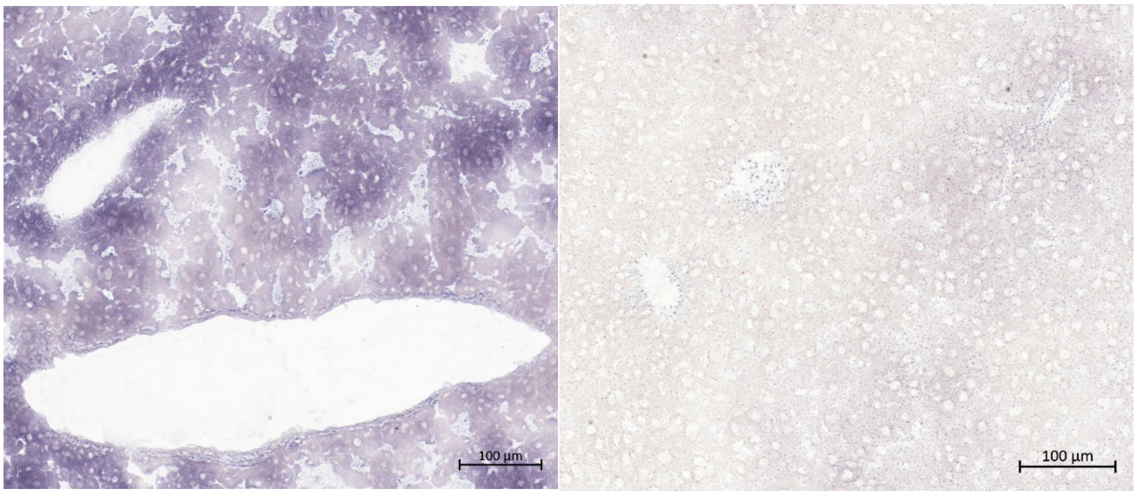

Supplementary Figure S2. The NBT signal for GDH activity on liver sections from Hep-*Glud1*$^{-/-}$ mice reveals the background signal of the assay. Control-*lox* and Hep-*Glud1*$^{-/-}$ mice were sacrificed and tissues were collected after 6 hours of fasting. The thickness of the liver cryosection for the NBT assay was at 7 µm. For the NBT assay, liver samples from Control-*lox* (**A**) and Hep-*Glud1*$^{-/-}$ mice (**B**) were incubated in a reaction buffer composed of 10% polyvinyl alcohol dissolved in PBS buffer (pH=8.0). The assay buffer contained 4 mM glutamate, 1.5 mM NAD$^+$, 1 mM ADP, 5 mM Nitro-Blue Tetrazolium salt, and 0.32 mM phenazine methosulfate. The incubation time was set to 30 min at 37°C. Scale bar=100 µm.

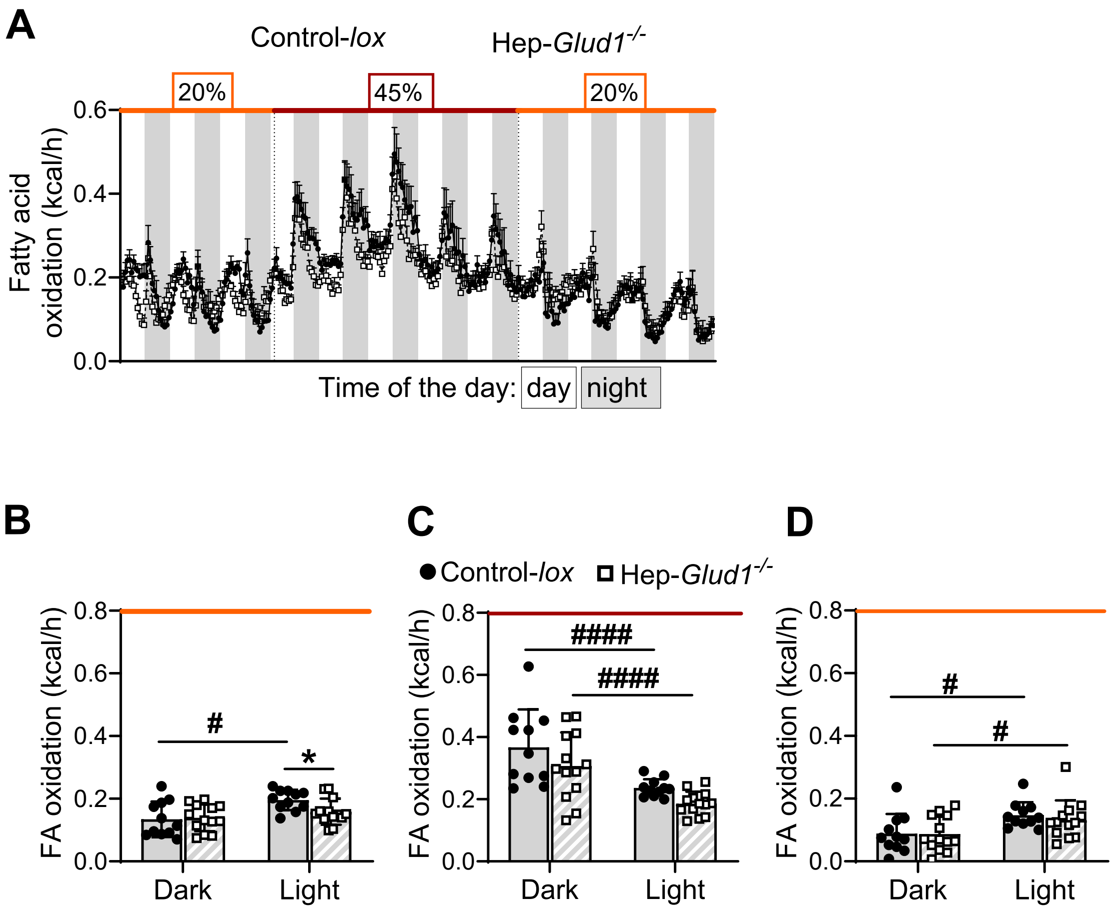

Supplementary Figure S3. Fatty acid oxidation changes in Control-*lox* and Hep-*Glud1*$^{-/-}$ mice fed different protein diet. Hourly fatty acid oxidation of Control-*lox* and Hep-*Glud1*$^{-/-}$ mice fed 20% protein diet (orange), then switched to 45% diet (dark red) and switched back to 20% protein diet (**A**). Average fatty acid oxidation in the dark and light phases during the initial 20% protein diet (**B**), during the second day of the 45% protein diet (**C**), and at the last day after the switch back to 20% protein diet (**D**). Data are shown as mean ± SD. Differences were calculated using repeated measures Two-way ANOVA followed by uncorrected Fisher's LDS test where *p< 0.05 for Control-lox vs Hep-*Glud1*$^{-/-}$, and #p< 0.05 for Dark vs Light within the same genotype.

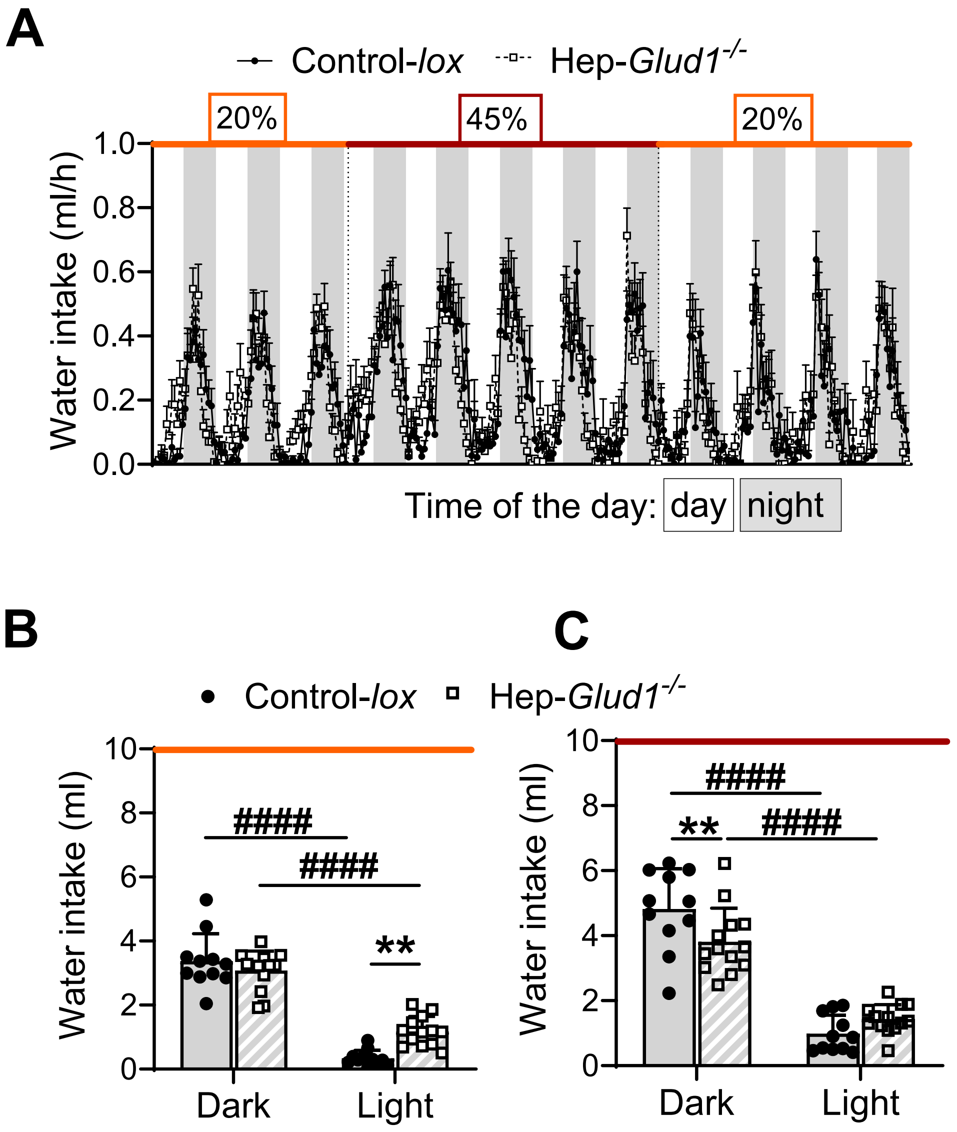

Supplementary Figure S4. Water intake consumption in Control-*lox* and Hep-*Glud1*$^{-/-}$ mice changes across different protein diets. Hourly water consumption for Control-*lox* and Hep-*Glud1*$^{-/-}$ mice fed 20% protein diet (orange), switched to 45% protein diet (dark red) and switched back to 20% protein diet (**A**). Average water intake during the dark and light phases of the initial 20% protein diet (**B**) and during the 4-day period fed 45% protein diet (**C**). Data are shown as mean ± SD. For B and C, differences were calculated as repeated measures Two-way ANOVA followed by uncorrected Fisher's LSD test where $*p < 0.05$ for Control-*lox* vs Hep-*Glud1*$^{-/-}$ and $\#p < 0.05$ for Dark vs Light within the same genotype.
